# Various radiological findings in patients with COVID-19: A case series

**DOI:** 10.1016/j.amsu.2021.01.030

**Published:** 2021-01-22

**Authors:** Theresia Riawati, Wikan Indrarto, Aditya Rifqi Fauzi, William Widitjiarso, Gunadi

**Affiliations:** a Department of Radiology, Panti Rapih General Hospital, Yogyakarta, 55233, Indonesia; b Department of Child Health, Panti Rapih General Hospital, Yogyakarta, 55233, Indonesia; c Faculty of Medicine, Public Health and Nursing, Universitas Gadjah Mada/Dr. Sardjito Hospital, Yogyakarta, 55281, Indonesia

**Keywords:** Abnormality findings, COVID-19, Ground glass opacity, Plain chest X-ray, Thoracic CT scan

## Abstract

**Introduction:**

Radiological evaluation of suspected COVID-19 patients is required for early detection of thoracic involvement, particularly in emergency units, while waiting for definitive diagnosis by real-time reverse transcription polymerase chain reaction (RT-PCR). Here, we report a case series of CXR findings in Indonesian patients with COVID-19 in our institution.

**Presentation of cases:**

We included 7 patients with COVID-19 confirmed by RT-PCR, including 4 females and 3 males, with ages ranging from 36 to 71 years. All patients showed abnormal findings on CXR when admitted to the hospital, except one, composed of ground glass opacity (GGO) (n = 1), consolidation (n = 3), and both (n = 2). Both and one side of the lung were affected in three and three (left side = 2; right side = 1) patients, respectively. Pneumonia degrees of mild, moderate and severe were observed in three, one, and two patients, respectively. All patients eventually recovered.

**Discussion:**

CXR is the most common radiological examination for patients with respiratory disorders, including COVID-19, and it is readily available in almost all health care facilities. The imaging manifestation of COVID-19 is similar to viral pneumonia but also has its own characteristics, including GGO, consolidation, multiple plaque shadows, and interstitial changes that are mostly seen in peripherals and subpleural areas, as well as shadow infiltration in both lungs.

**Conclusion:**

CXR showed various abnormality findings in patients with COVID-19, including the type, location, and degree of pneumonia. Moreover, CXR is considered more effective and useful for initial screening and follow-up of the progress of patients with COVID-19.

## Highlights

• CXR is the most common radiological examination for patients with respiratory disorders, including COVID-19.
• CXR is considered more effective and useful for initial screening and follow-up of the progress of patients with COVID-19.
• CXR shows various abnormality findings in patients with COVID-19, including the type, location, and degree of pneumonia.

## 1 Introduction

Severe Acute Respiratory Syndrome Coronavirus 2 (SARS-CoV-2), which causes Coronavirus Disease 2019 (COVID-19), has become a global pandemic that infected nearly 11 million people and claimed 523,011 lives as of July 4, 2020 [1,2].

The first two cases in Indonesia were declared on March 2, 2020. Since it was first announced in Indonesia, COVID-19 cases have increased rapidly over time, thus requiring continued attention. On July 5, Indonesia recorded 63,749 COVID-19 infections [3]. Until now, radiological examinations have an important role in the management of COVID-19, especially for screening tests, working diagnosis and monitoring pneumonia [4,5].

Plain chest X-rays (CXR) and computerized tomography (CT) scans are key radiological or imaging examinations in confirming COVID-19 diagnosis. CT scans are reported to be more sensitive in diagnosing COVID-19 than CXR [6]. However, its use is limited due to its expense and is not widely available, especially in developing countries. Therefore, CXR is considered more effective and useful for the initial screening and follow-up of patients with COVID-19 [7,8]. Moreover, radiological evaluation of suspected COVID-19 patients is required for early detection of thoracic involvement, particularly in emergency units, while waiting for definitive diagnosis by real-time reverse transcription polymerase chain reaction (RT-PCR) [9]. In Indonesia, there is no consensus on the use of chest CT scans for COVID-19 cases, but it depends on the availability of tools, facilities, and human resources in most hospitals. Chest CT scanning is not recommended for screening tests and in patients with mild and asymptomatic symptoms but is indicated in patients with negative RT-PCR; however, they show worsening clinical signs and severe pneumonia with complications. Here, we report a case series of CXR findings in Indonesian patients with COVID-19. This study has been reported in line with PROCESS criteria [10].

## 2 Presentation of cases

There were 7 patients diagnosed with COVID-19 according to positive RT-PCR results in our hospital. They consisted of 4 females and 3 males, with ages ranging from 36 to 71 years.

**Fig. 1:**
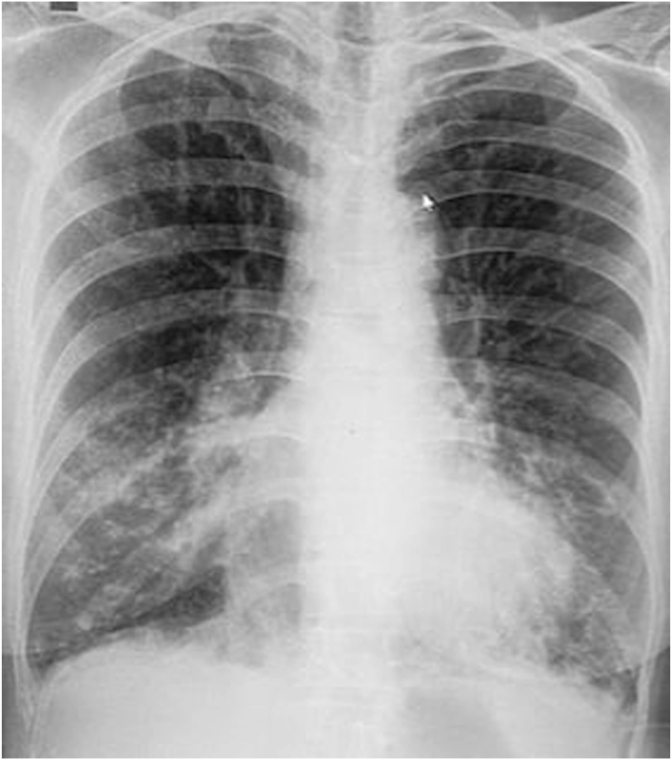
Plain chest X-ray showed bilateral consolidation in the basal lung.

**Case 1:** A 59-year-old female presented with cough with phlegm, runny nose, fever, and shortness of breath 3 days before admission. The patient was taking self-medication using decongestant drugs, but her complaints did not improve. There was no history of traveling to areas with COVID-19 local transmission. A CXR was performed, and the results showed bilateral consolidation in the basal lung (Fig. 1). The patient was then hospitalized with a diagnosis of bronchial asthma. Later, an RT-PCR test for COVID-19 was performed, and the results were positive. During treatment, the patient received antibiotic therapy, namely, intravenous ceftazidime 1 gr and intravenous levofloxacin 500 mg, while she also received medication for her asthma.

**Fig. 2:**
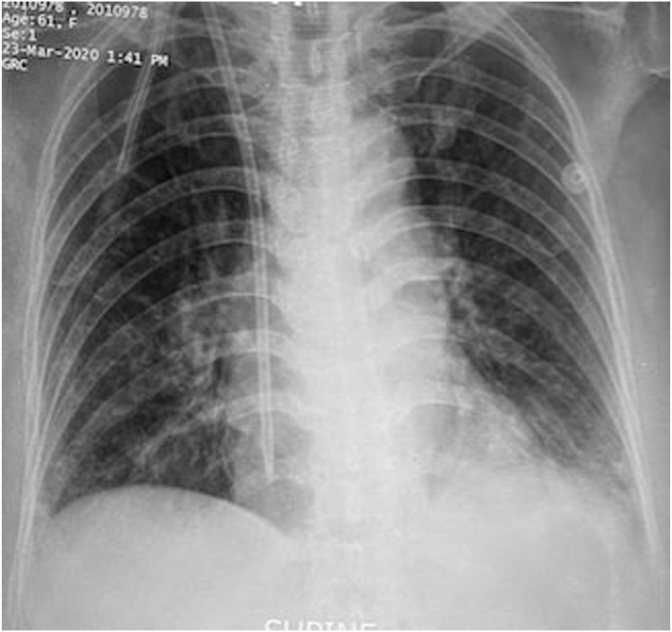
Plain chest X-ray revealed consolidation in the basal lung sinistra.

**Case 2:** A 61-year-old female presented with fever, shortness of breath, and cough. Complaints of shortness of breath felt worse starting 1 day before admission. The patient was brought to the emergency room, and a CXR was performed. The result showed consolidation in the basal lung sinistra (Fig. 2). The patient had comorbid diabetes mellitus and chronic renal failure. RT-PCR swab tests were performed, and the results were positive. The patient was diagnosed with COVID-19, chronic kidney disease and diabetes mellitus. After admission, the patient received antibiotics and antiviral therapy based on the COVID-19 Prevention and Control guidelines by the Indonesian Ministry of Health, namely, oral hydroxychloroquine 200 mg twice daily and oral oseltamivir 75 mg twice daily, oral moxifloxacin 400 mg, and intravenous meropenem 1gr thrice daily for his COVID-19, while she also received medication for her chronic renal disease, hypertension, and diabetes.

**Fig. 3:**
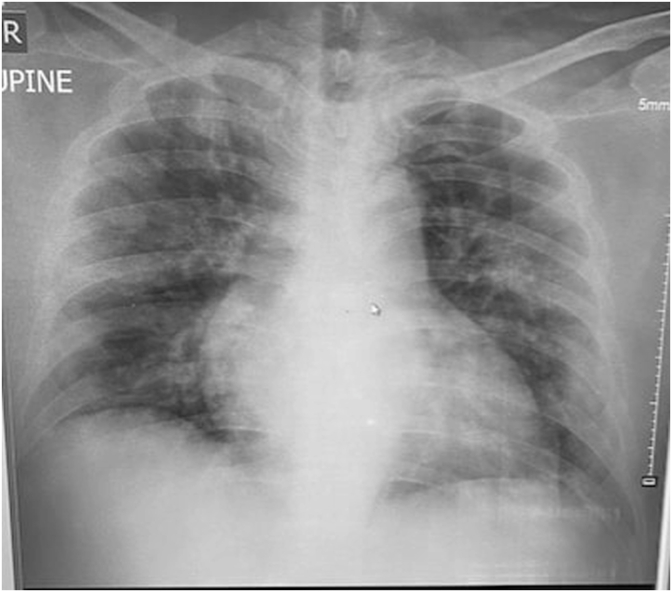
Plain chest X-ray presented GGOs in the periphery of the left lung and consolidation in the bilateral parahilar and paracardial regions of the lung.

**Case 3:** A 40-year-old male with complaints of fever 11 days before admission, accompanied by cough, runny nose, shortness of breath, dizziness, and nausea, came to our hospital. The patient had a history of asthma, diabetes and hypertension that was controlled with routine medication. The patient had comorbidities of diabetes mellitus and hypertension. A CXR was performed, and the results showed ground glass opacities (GGOs) in the periphery of the left lung and consolidation in the bilateral parahilar and paracardial regions of the lung (Fig. 3). RT-PCR swab examination was done, and the result was positive for COVID-19. After admission, the patient received antibiotics and antiviral therapy based on the COVID-19 Prevention and Control guidelines by the Indonesian Ministry of Health, namely, oral hydroxychloroquine 200 mg twice daily and oral oseltamivir 75 mg twice daily, intravenous meropenem 1gr thrice daily, intravenous levofloxacin 500 mg, and intravenous imipenem 500 mg four times a day for his COVID-19, while also receiving medication for his diabetes and hypertension.

**Fig. 4:**
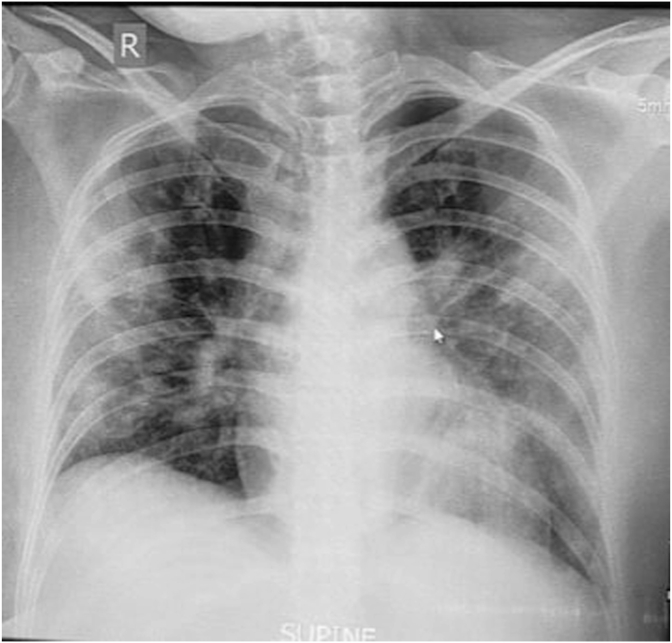
Plain chest X-ray showed the appearance of GGO in the periphery of both the lung and multifocal consolidation parahilar and paracardial in the right lung.

**Case 4:** A 48-year-old female presented with fever 10 days before admission, accompanied by cough and vomiting. Her husband had a history of traveling from a local transmission area. The patient had comorbid diabetes mellitus. A CXR was done, showing the appearance of GGO in the periphery of both lungs and multifocal consolidation parahilar and paracardial in the right lung (Fig. 4). RT-PCR for COVID-19 was done, and the results were positive. After admission, the patient received antibiotics and antiviral therapy based on the COVID-19 Prevention and Control guidelines by the Indonesian Ministry of Health, namely, oral lopinavir/ritonavir 400mg/100 mg once daily, intravenous levofloxacin 750 mg once daily, and oral hydroxychloroquine 200 mg twice daily, and intravenous meropenem 1gr thrice daily for her COVID-19 diagnosis, while also receiving medication for the possibility of bacterial infection.

**Fig. 5:**
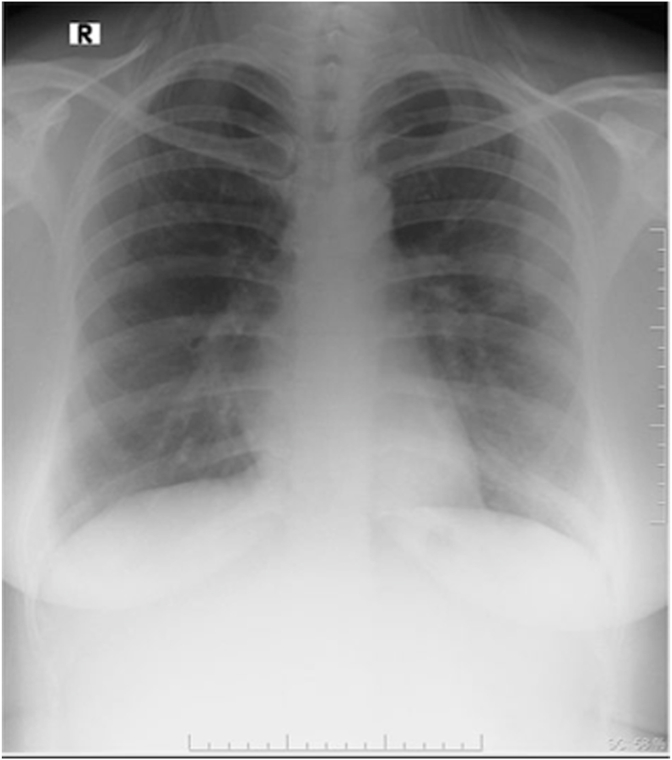
Plain chest X-ray displayed GGO in the periphery of the left lung.

**Case 5:** A 36-year-old female with the chief complaint of fever 4 days before admission. There was no history of contact with suspected or confirmed COVID-19 patients. A CXR showed GGO in the periphery of the left lung. RT-PCR for COVID-19 was done, and the results were positive. After admission, the patient received antibiotics and antiviral therapy based on the COVID-19 Prevention and Control guidelines by the Indonesian Ministry of Health, namely, oral lopinavir/ritonavir 400 mg/100 mg once daily, oral hydroxychloroquine 200 mg twice daily, intravenous azithromycin 500 mg once daily, and oral oseltamivir 75 mg twice daily (Insert Fig. 5 here).

**Fig. 6:**
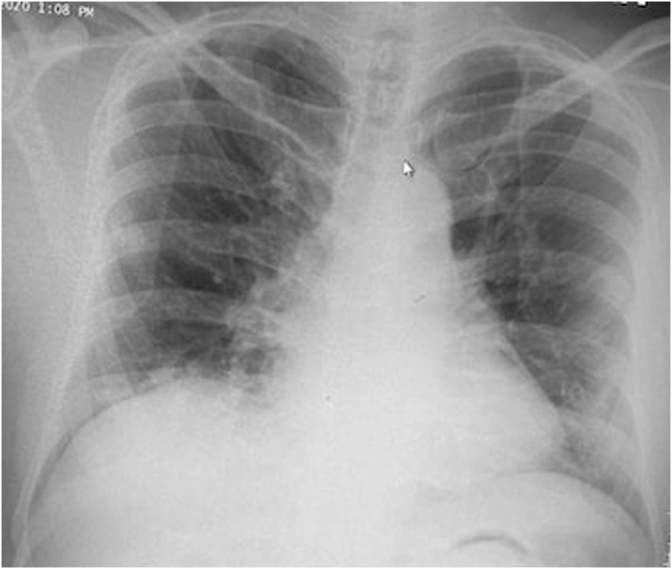
Plain chest X-ray demonstrated consolidation in the basal right lung.

**Case 6:** A 57-year-old male came to our hospital without complaints. He brought the RT-PCR COVID-19 swab results, which showed that he was positive. He had a history of traveling to the local transmission area. The patient had no comorbidities. A CXR revealed consolidation in the basal right lung (Fig. 6). Patient was hospitalized, and after admission, she received antibiotics and antiviral therapy based on the COVID-19 Prevention and Control guidelines by the Indonesian Ministry of Health, namely, oral azithromycin 500 mg once daily, oral chloroquine 150 mg twice daily, oral methisoprinol 1000 mg thrice daily, and oral oseltamivir 75 mg twice daily.

**Fig. 7:**
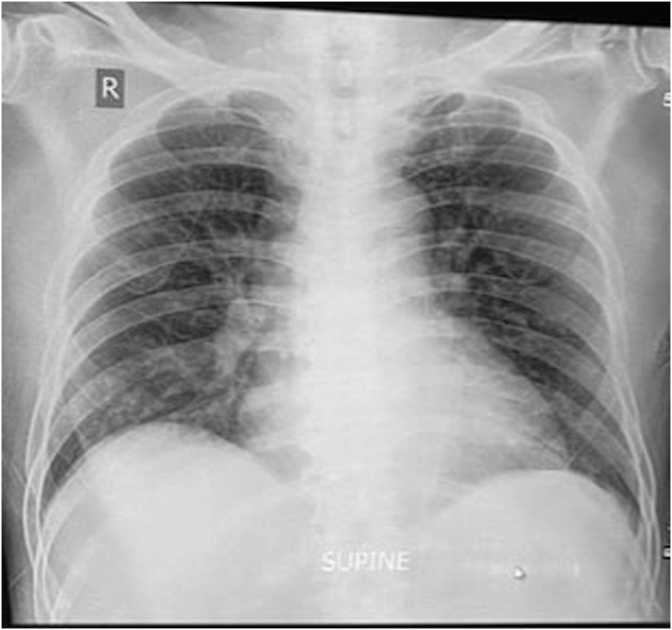
Plain chest X-ray was within normal limits.

**Case 7:** A 71-year-old male presented with a complaint of pain in his right leg, and the patient asked to be treated. The patient had a history of living with his children who did not comply with the COVID-19 prevention health protocol. His CXR showed cardiomegaly with configuration of left ventricle hypertrophy (Fig. 7). The patient had a history of diabetes mellitus. The patient underwent an RT-PCR swab examination for COVID-19, and the results were positive. He was treated with a diagnosis of COVID-19, anemia and diabetes mellitus, and diabetic ulcer on his left foot. After admission, the patient received antibiotics and antiviral therapy based on the COVID-19 Prevention and Control guidelines by the Indonesian Ministry of Health, namely, intravenous ceftazidime 1 gr thrice daily, oral azithromycin 500 mg twice daily, oral chloroquine 150 mg twice daily, and oral oseltamivir 75 mg twice daily, while he also received therapy for his diabetes and diabetic ulcer.

## 3 Discussion

To date, confirmation of the diagnosis of COVID-19 requires viral nucleic acid detection from throat swabs using RT-PCR, although this test is considered only specific but not sensitive. Current studies have shown that lung imaging can manifest earlier than clinical symptoms. Therefore, detecting the disease quickly and accurately is of great significance, and imaging is playing a key role in preclinical screening [6,11].

CXR is the most common radiological examination for patients with respiratory disorders, including COVID-19 [12]. Since it is readily available in almost all health care facilities, particularly in developing countries, and less expensive, CXR is considered more effective and useful for initial screening and follow-up of the progress of patients with COVID-19 [7,8].

The typical CXR in patients with COVID-19 is GGO. In addition, consolidation is usually multifocal, peripheral and bilateral, but in the early stages of the disease, it can be unifocal and is most often seen in the inferior lobe of the right lung. Pleural effusion and hilar lymphadenopathy are rare. This bilateral pulmonary involvement differentiates COVID-19 from bacterial pneumonia [12]. These findings are compatible with our patients.

Moreover, the degree of pneumonia based on CXR consists of no abnormality/normal, focal-unilateral/mild, bilateral/moderate focal, and multifocal-bilateral/severe [13]. Most of our patients with COVID-19 showed bilateral, multifocal, and severe pneumonia.

The imaging manifestation of COVID-19 is similar to viral pneumonia but also has its own characteristics, such as multiple plaque shadows and interstitial changes that are mostly seen in peripherals and subpleural areas, as well as shadow infiltration in both lungs. In severe cases, it can appear as a consolidation with a “white lung” image [13]. Commonly seen patterns are GGO, with ill-defined margins, air bronchograms, smooth or irregular interlobular or septal thickening, and thickening of the adjacent pleura, with predominance in the right lower lobe [14]. These findings are quite similar to the radiographic images of SARS infection [15], except SARS shows more unifocal rather than bilateral involvement in COVID-19. Middle-East Respiratory Syndrome (MERS) pneumonia also shares similarities in subpleural and basilar airspace lesions, with extensive GGO and consolidation [16].

Even though CXR is less sensitive than CT scanning to diagnose COVID-19, it has a significant role in the management of the outbreak [17,18]. Additionally, CT scans have a very low specificity to detect the peculiar findings of pneumonia due to COVID-19 [18,19]. Other advantages of CXR over CT scans are as follows: 1) it is easier to operate the X-rays, particularly the procedure of disinfection that should be performed after each examination, and 2) it can be conducted at the bedside of patients, therefore minimizing the cross-infection risk in the radiology department [9].

## 4 Conclusions

CXR shows various abnormality findings in patients with COVID-19, including the type, location, and degree of pneumonia. Moreover, CXR is considered more effective and useful for initial screening and follow-up of the progress of patients with COVID-19.

## Consent

Written informed consent was obtained from the patient for publication of this case report and accompanying images. A copy of the written consent is available for review by the Editor-in-Chief of this journal on request.

## Provenance and peer review

Not commissioned, externally peer-reviewed.

## Declaration of competing interest

No potential conflict of interest relevant to this article was reported.

## Registration of research studies

researchregistry5709.
